# Single centre experience of the replacement of ascending aorta with different types of valve-containing conduit

**DOI:** 10.1186/1749-8090-8-S1-O9

**Published:** 2013-09-11

**Authors:** I Andraloits, V Shumavets, A Shket, S Spiridonau, S Kurganovich, L Baraukova, Y Osrtovsky

**Affiliations:** 1 Cardiac Surgery Department, Belarus Cardiology Centre, Minsk, Belarus

## Background

To compare immediate postoperative surgery results in patients after replacement of ascending aorta and aortic valve with various modifications of valve-containing conduit.

## Methods

Replacement of ascending aorta and aortic valve from 2009 till 2013 was performed in 194 patients (18; 9,3% redo) with pathology of the aortic root. In 19 cases BioValsalva conduit was used (9,3%); in 15 pts (7,7%) allografts and in 6 (3,1%) stentless bioprosthesis were used with “full-root” technique; vascular graft conduits containing stented bioprosthesis in 16 pts (8,2%) or different types of mechanical valve in 139 (71,6%) were used with modification of Bentall procedure. The average age of the patients was 55,7±12,2 years, 158 men (81,4%). 34 patients (17,5%) underwent emergency surgery due to acute dissecting of the thoracic aorta.

## Results

Hemiarch operation were performed in 8 cases, aortic arch complete replacement – in 20 cases; concomitant coronary artery bypass (CABG) – in 33 cases (17%); concomitant correction on mitral valve – in 34 cases (35%). In-hospital mortality were 7,8 % (n=15, 95%CI 5,7%–9,1%) and did not depend on the type of the conduit used. Mortality in emergency and in redo was not significantly higher 12,5% vs 6,9% (χ2–1,05, p=0,2).Cross-clamp and CPB time significantly differed for various types conduits (p < 0,05). Frequency of reopen due to postoperative bleeding did not differ between groups averaging 8,2% (n=16, χ2–3,31, p=0,93). In the BioValsalva group a smaller prosthesis diameters (21-23 mm) were used often (χ2–36,79, p=0,012). However effective opening area did not significantly differ for different types of conduits with mean iEOA 1.2±0,18 cm2/m2 (p=0,09).

## Conclusion

The results show that BioValsalva prostheses are noninferior to other conduits used if choosing smaller valve diameter. Further observation of these patients is required in order to assess long-term results and determining optimum type of valve-containing conduit.

